# Disturbances in PP2A methylation and one-carbon metabolism compromise Fyn distribution, neuritogenesis, and APP regulation

**DOI:** 10.1074/jbc.RA120.016069

**Published:** 2021-01-07

**Authors:** Goce Taleski, Diana Schuhmacher, Henry Su, Jean-Marie Sontag, Estelle Sontag

**Affiliations:** 1 School of Biomedical Sciences and Pharmacy, University of Newcastle, Callaghan, NSW, Australia; 2 Department of Pathology, UT Southwestern Medical Center, Dallas, Texas, USA

**Keywords:** protein phosphatase 2 (PP2A), Fyn, one-carbon metabolism, homocysteine, protein methylation, tyrosine-protein kinase (tyrosine kinase), signaling, amyloid precursor protein (APP), Alzheimer's disease, neurite outgrowth, 3-DZA, 3-deazaadenosine, Aβ, amyloid-β, AD, Alzheimer's disease, anti-HA, anti-hemagglutinin, APP, amyloid precursor protein, EV, empty vector, FBS, fetal bovine serum, FD, folate-deficient, HA, hemagglutinin, Hcy, homocysteine, HTL, Hcy thiolactone, LCMT1, leucine carboxyl methyltransferase 1, MβCD, methyl-β-cyclodextrin, N2a, Neuro-2a, OA, okadaic acid, PP2A, protein phosphatase 2A, PP2Ac, catalytic “C” subunit of PP2A, pSFK, phosphorylated SFK, SFKs, Src family kinases

## Abstract

The nonreceptor protein tyrosine kinase Fyn and protein Ser/Thr phosphatase 2A (PP2A) are major multifunctional signaling molecules. Deregulation of Fyn and altered PP2A methylation are implicated in cancer and Alzheimer's disease (AD). Here, we tested the hypothesis that the methylation state of PP2A catalytic subunit, which influences PP2A subunit composition and substrate specificity, can affect Fyn regulation and function. Using Neuro-2a (N2a) neuroblastoma cell models, we first show that methylated PP2A holoenzymes containing the Bα subunit coimmunoprecipitate and copurify with Fyn in membrane rafts. PP2A methylation status regulates Fyn distribution and Fyn-dependent neuritogenesis, likely in part by affecting actin dynamics. A methylation-incompetent PP2A mutant fails to interact with Fyn. It perturbs the normal partitioning of Fyn and amyloid precursor protein (APP) in membrane microdomains, which governs Fyn function and APP processing. This correlates with enhanced amyloidogenic cleavage of APP, a hallmark of AD pathogenesis. Conversely, enhanced PP2A methylation promotes the nonamyloidogenic cleavage of APP in a Fyn-dependent manner. Disturbances in one-carbon metabolic pathways that control cellular methylation are associated with AD and cancer. Notably, they induce a parallel loss of membrane-associated methylated PP2A and Fyn enzymes in N2a cells and acute mouse brain slices. One-carbon metabolism also modulates Fyn-dependent process outgrowth in N2a cells. Thus, our findings identify a novel methylation-dependent PP2A/Fyn signaling module. They highlight the underestimated importance of cross talks between essential metabolic pathways and signaling scaffolds that are involved in normal cell homeostasis and currently being targeted for cancer and AD treatment.

Fyn is a member of the Src family kinases (SFKs) of nonreceptor protein tyrosine kinases that modulate a plethora of key cellular functions, including growth, survival, adhesion, migration, and differentiation (1). Expectedly, deregulation of these important signaling enzymes is associated with numerous pathological conditions, including cancer (2). Of particular interest, deregulation of Fyn also participates in Alzheimer's disease (AD) pathogenesis. In AD, the abnormal accumulation of amyloid-β (Aβ) peptides derived from enhanced β- and γ-secretase cleavage of amyloid precursor protein (APP) is believed to be a key event in initiating hallmark neurodegenerative cascades (3). Pathological levels of oligomeric Aβ species are linked to aberrant overstimulation of postsynaptic Fyn-dependent signaling pathways, ultimately leading to impaired synaptic and cognitive functions, and neurotoxicity in several animal models of AD (4). Overactivation of Fyn also induces an abnormal elevation of APP phosphorylated at Tyr^682^, causing its mistrafficking and missorting in neurons, with important consequences for Aβ production (5). Inhibition of Fyn activity can counteract memory and synaptic deficits in AD mice (6), further demonstrating the essential link between Fyn deregulation and AD pathogenic pathways.

SFKs transduce signals from a variety of receptors *via* their ability to form complexes with numerous cytoskeletal and signaling proteins at the plasma membrane (1, 2). The spatial localization and signaling activity of SFKs is tightly controlled by endocytic trafficking (7, 8). In neuronal cells, the myristoylated and palmitoylated Fyn kinase is preferentially enriched and activated in sphingolipid- and cholesterol-enriched plasma membrane microdomains traditionally referred to as lipid or membrane rafts (9, 10, 11). These specialized microdomains serve a key role in cell signaling and function by compartmentalizing and regulating interactions of key membrane proteins (12). For instance, activation of raft-associated Fyn stimulates neurite outgrowth (13, 14, 15) and regulates the targeting of APP to lipid rafts (11). Notably, membrane microdomain switching is a key determinant of APP processing and function (16, 17, 18, 19). Under normal physiological conditions, a majority of APP undergoes proteolytic processing by α-secretase, which precludes Aβ formation and generates neurotrophic-secreted soluble amyloid precursor protein α (sAPPα) fragments. There is strong support that the α-secretase cleavage of APP preferentially occurs in nonraft membrane microdomains, while its amyloidogenic processing primarily takes place in lipid rafts (17, 18, 19).

Another major signaling molecule deregulated in cancer (20) and AD (21) is protein Ser/Thr phosphatase 2A (PP2A). The “PP2A family” encompasses multimeric enzymes with the typical mammalian holoenzyme being composed of a catalytic “C” subunit (PP2Ac) associated with a scaffolding “A” subunit and a variable regulatory “B” subunit. PP2A biogenesis, stability, and substrate specificity can be modulated by leucine carboxyl methyltransferase 1 (LCMT1)-dependent methylation of PP2Ac on the Leu309 residue; conversely, PP2Ac is demethylated by the methylesterase, PME1 (22). We have previously reported that reduced PP2A methylation is associated with a loss of PP2A/Bα holoenzymes that contain the regulatory Bα (or PPP2R2A) subunit and altered dephosphorylation of PP2A/Bα substrates, including APP phosphorylated at the Thr^668^ site, in Neuro-2a (N2a) cells and *in vivo* (23, 24). Phosphorylation of APP at Thr^668^ and Tyr^682^ regulates APP interactions (25), subcellular localization, processing, and function, so that abnormally enhanced phosphorylation of APP in AD likely contributes to APP dysfunction (3, 5). PP2A methylation becomes downregulated in AD and after alterations in one-carbon metabolism in cells and *in vivo* (21). Disturbances in one-carbon metabolism that promote toxic elevation of plasma homocysteine (Hcy) and its oxidized derivatives and inhibition of cellular methylation are strongly associated with AD (26, 27) and cancer (28).

In this study, using neuroblastoma N2a cell models, we show that intact PP2A methylation is essential for the formation of PP2A/Bα-Fyn protein complexes and their codistribution in membrane rafts. Altered PP2A methylation promotes a redistribution of Fyn and inhibits Fyn-dependent neuritogenesis. It affects the compartmentalization of Fyn and APP in membrane microdomains, which regulates APP processing. Manipulations of one-carbon metabolism that modulates PP2A methylation state also affect Fyn distribution. Our findings identify a novel mechanism of regulation of Fyn at the crossroads of metabolism and signaling.

## Results

### PP2A coimmunoprecipitates with Fyn in a methylation-dependent manner

**Figure 1:**
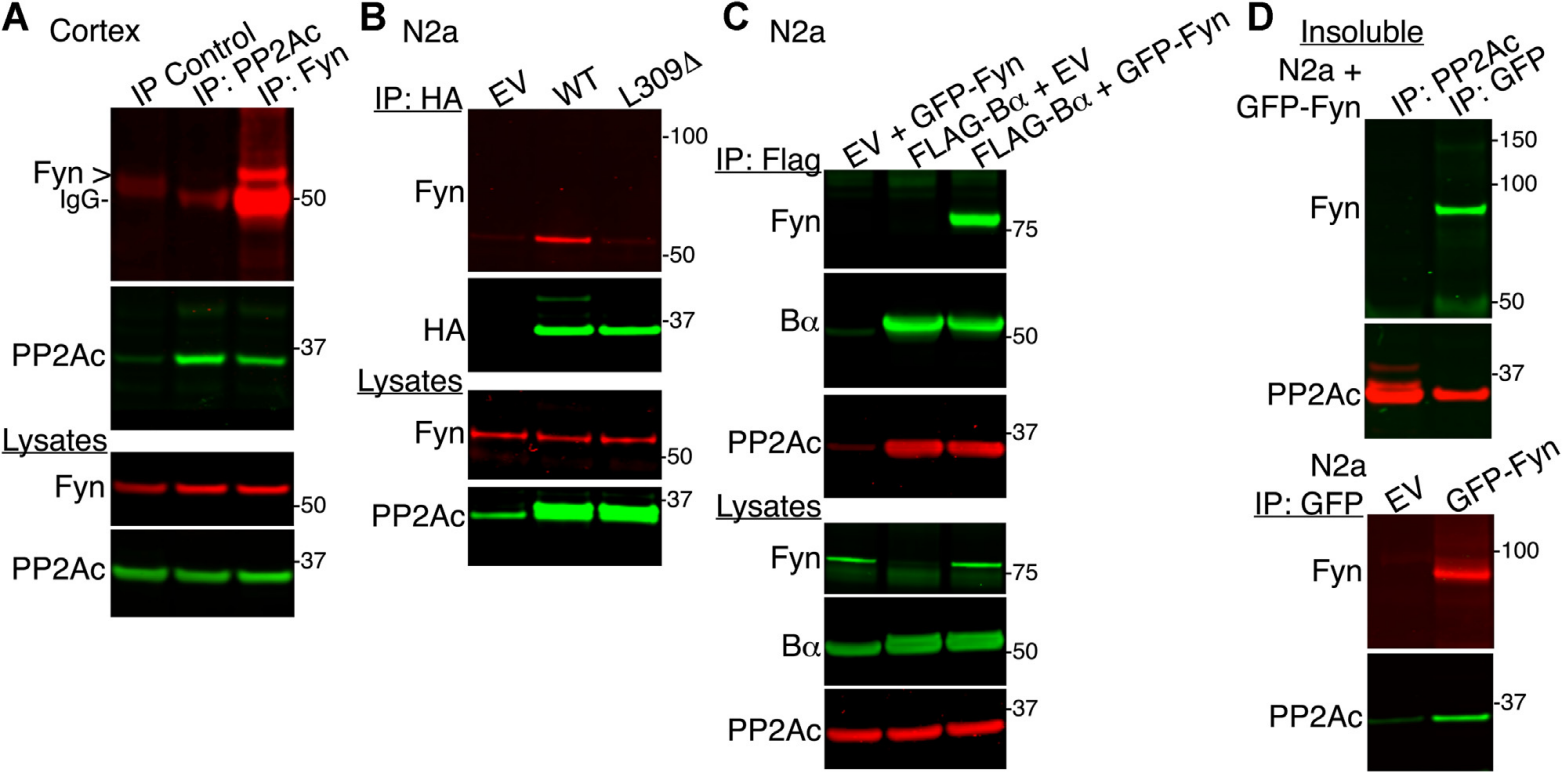
**Methylated PP2A/Bα enzymes coimmunoprecipitate with Fyn.***A*, coimmunoprecipitation of endogenous PP2Ac and Fyn from total mouse cortical lysates. *B*, HA immunoprecipitates and total lysates from N2a cells stably expressing HA-tagged PP2Ac (WT), the methylation-incompetent L309Δ PP2Ac mutant (L309Δ), or empty vector (EV) were immunoblotted for Fyn, HA, or PP2Ac. *C*, flag immunoprecipitates and total lysates from N2a cells transfected with the indicated plasmids were immunoblotted for Fyn, PP2Ac, and PP2A-Bα. *D*, the whole NP-40 detergent-insoluble fraction prepared from GFP–Fyn–expressing N2a cells was divided into two equal parts. GFP or PP2Ac immunoprecipitates were prepared from each aliquot and analyzed for the presence of Fyn and PP2Ac (*top* panel). No Fyn or PP2Ac was found in control GFP immunoprecipitates carried out in EV-transfected, compared with, GFP–Fyn–expressing N2a cells (*bottom* panel). Representative blots from 3 separate experiments are shown in panels *A*–*D*. HA, hemagglutinin; IgG, immunoglobulin; PP2A, PP2A, phosphatase 2A; PP2Ac, catalytic “C” subunit of PP2A; N2a, Neuro-2a.

Based on the reported *in vitro* binding of PP2Ac to Src (29), an SFK structurally closely related to Fyn, we first assessed the existence of PP2A–Fyn protein complexes using a series of coimmunoprecipitation assays. Western blot analyses showed that endogenous PP2Ac was present in Fyn immunoprecipitates prepared from mouse cortical homogenates (Fig. 1*A*). However, endogenous Fyn was not detected in corresponding PP2Ac immunoprecipitates. Although these data suggest that PP2A is a major Fyn interacting protein and Fyn is a minor interacting partner of PP2Ac, it is worth noting that anti-PP2Ac subunit antibodies are ineffective at quantitatively pulling down native heterotrimeric PP2A holoenzymes, the predominant species in mammalian cells (30). To circumvent this problem, anti-hemagglutinin (anti-HA)–conjugated beads were used to effectively immunoprecipitate PP2A holoenzymes from well-characterized N2a cell lines stably expressing hemagglutinin (HA)-tagged WT PP2Ac or the L309Δ PP2Ac mutant, as described previously (23). In contrast to the WT, the L309Δ mutant is methylation incompetent and unable to associate with regulatory B-type subunits in N2a (31) and other cell lines (22). Cells transfected with an empty vector (EV) were used as controls. Western blot analyses revealed that endogenous Fyn was present in HA–PP2Ac immunoprecipitates prepared from WT but not L309Δ-expressing or control N2a cell lysates (Fig. 1*B*), suggesting that Fyn preferentially associates with B-containing methylated PP2A holoenzymes. To confirm the existence of PP2A/Bα–Fyn protein complexes, immunoprecipitates were prepared from N2a cells coexpressing Flag–Bα and GFP–Fyn. We chose this approach based on the lack of suitable antibodies to pull down PP2A/Bα holoenzymes and the close proximity of endogenous Bα, Fyn, and immunoglobulins on gels. GFP–Fyn was clearly concentrated with the Bα subunit in Flag–Bα immunoprecipitates prepared from these cells but was absent in parallel control immunoprecipitations (Fig. 1*C*). The concomitant presence of PP2Ac in these immunoprecipitates further confirmed that PP2A/Bα holoenzymes were successfully pulled down under our experimental conditions.

In agreement with the preferential targeting of Fyn to membrane rafts, Fyn is enriched in detergent-insoluble fractions obtained after lysing tissue or cells with the nonionic detergent, NP-40 (32). Typically, NP-40 detergent-insoluble fractions contain highly insoluble lipid rafts and cytoskeletal components, whereas the detergent-soluble fraction largely consists of cytosolic proteins and extracted proteins “less tightly” attached to membranes. Endogenous PP2Ac coimmunoprecipitated with Fyn in NP-40 detergent-insoluble fractions prepared from GFP–Fyn–transfected but not EV-transfected N2a cells (Fig. 1*D*), supporting the existence of membrane-associated PP2A–Fyn protein complexes. Together, these findings indicate that methylation-dependent PP2A/Bα holoenzymes are important for the formation of PP2A–Fyn protein complexes.

### PP2A methylation state affects the levels of membrane-associated Fyn in N2a cells

**Figure 2:**
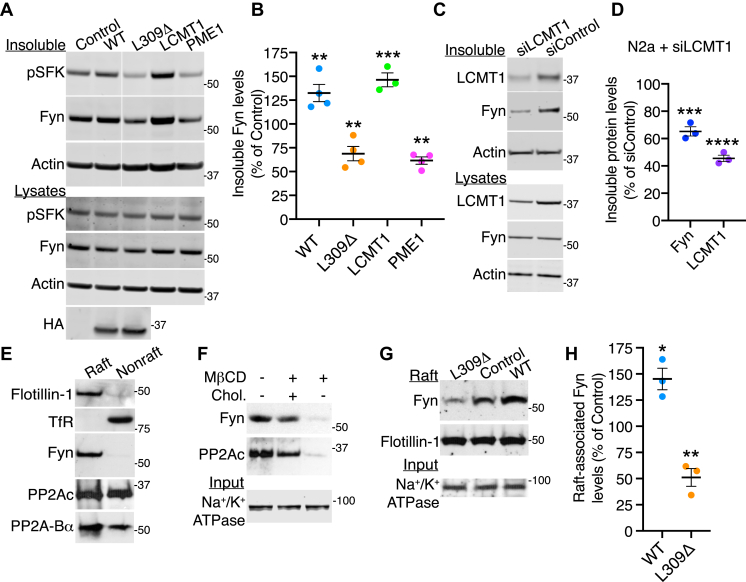
**Changes in PP2A methylation influence the discrete membrane distribution of Fyn in N2a cells.***A*, representative immunoblots of pY416-SFK (pSFK), Fyn, and actin in total lysates and NP-40 detergent–insoluble fractions prepared from N2a cells stably expressing WT PP2Ac, the L309Δ PP2Ac mutant, LCMT1, PME1, or empty vector (control). Panels in detergent-insoluble fractions originated from the same blot. *B*, quantification of Fyn levels in NP-40 detergent–insoluble fractions from these cells. Data (mean ± SEM from *n* = 3–4 independent experiments) were appraised using one-way ANOVA (F (4, 14) = 33.44; *p* < 0.0001) with Dunnett’s post hoc test. ∗∗*p* < 0.01, ∗∗∗*p* < 0.001, *versus* control. *C*, total lysates and NP-40 detergent–insoluble fractions purified from N2a cells transfected with a validated siRNA targeted to LCMT1 (siLCMT1) or a mismatch siRNA control (siControl) were analyzed by Western blotting for the presence of Fyn and LCMT1. *D*, Fyn and LCMT1 protein levels were decreased in detergent-insoluble fractions from siLCMT1 relative to siControl-transfected N2a cells. Data (mean ± SEM; *n* = 3 separate experiments) were analyzed using a student *t*-test. ∗∗∗*p* < 0.001, ∗∗∗∗*p* < 0.0001. *E*, representative immunoblots of PP2ABα and PP2Ac subunits, Fyn, flotillin-1, and transferrin receptor (TfR) in raft and nonraft membrane fractions purified from N2a cells. Similar results were obtained in three separate purifications. *F*, representative distribution of PP2Ac and Fyn in aliquots (15 μg) of raft fractions purified from N2a cells that were incubated for 15 min in a serum-deficient medium in the absence (−) or presence (+) of the cholesterol depletion agent MβCD or cholesterol (Chol). Total membrane fractions (input) from these cells were probed with an antibody against the membrane marker, sodium potassium adenosine triphosphatase (Na^+^/K^+^ ATPase). *G*, representative Western blot analysis of total membrane fractions (input) and rafts purified from EV-, WT-, or L309Δ-transfected N2a cells. *H*, relative levels of raft-associated Fyn were quantified in EV-, WT-, and L309Δ-expressing N2a cells. Data (mean ± SEM from *n* = 3 separate purifications) were analyzed using a student *t*-test. ∗*p* < 0.05; ∗∗*p* < 0.01, *versus* control. EV, empty vector; LCMT1, leucine carboxyl methyltransferase 1; N2a, Neuro-2a; MβCD, methyl-β-cyclodextrin; PP2A, PP2A, protein phosphatase 2A; PP2Ac, catalytic “C” subunit of PP2A.

In light of the importance of PP2A methylation and subunit composition for its membrane targeting (33) and interaction with Fyn (Fig. 1*B*) in N2a cells, we next assessed whether modulating PP2A methylation state has any influence on the steady-state distribution of Fyn. To that end, total lysates and Fyn-enriched NP40-detergent insoluble fractions were first prepared from our N2a cell models and comparatively analyzed by Western blotting for relative changes in endogenous Fyn levels. Compared with controls, detergent-insoluble Fyn levels were overall increased in WT-expressing N2a cells (Fig. 2, *A* and *B*), which display a proportional ∼30% increase in total cellular and membrane-associated PP2Ac amounts (33). In contrast to the WT, there was a decrease in insoluble Fyn levels in L309Δ-expressing N2a cells. In these cells, there is a ∼35 to 45% reduction of methylated PP2Ac and Bα subunit levels and concomitant accumulation of demethylated PP2Ac (23); these methylation deficits induce a pronounced loss of PP2A/Bα enzymes targeted to the plasma membrane (33). Further demonstration of the regulatory role of PP2A methylation in Fyn distribution was obtained by deregulating PP2A methyltransferase and methylesterase enzymes. Overexpression of LCMT1, which promotes a ∼30% increase in the steady-state levels of total and membrane-associated methylated PP2Ac and PP2A/Bα in N2a cells (23, 33), markedly enhanced the amounts of Fyn in detergent-insoluble fractions (Fig. 2, *A* and *B*). Conversely, those were reduced after overexpression of PME-1, which promotes the accumulation of demethylated PP2Ac, downregulation of PP2A/Bα, and concomitant decrease in membrane levels of methylated PP2A enzymes (23, 33). Notably, this reduction in detergent-insoluble Fyn levels was recapitulated by inducing partial, siRNA-mediated LCMT1 downregulation in N2a cells (Fig. 2, *C* and *D*). This submaximal reduction in LCMT1 levels is associated with a pronounced loss of methylated PP2Ac and PP2A/Bα (34), and membrane-associated LCMT1 and PP2Ac in N2a cells (33). Thus, our findings suggest that PP2A methylation state concomitantly affects the membrane targeting of PP2A/Bα and Fyn.

Parallel immunoblotting of total cell lysates confirmed that the differential enrichment of Fyn in NP-40 detergent-insoluble fractions in our cell models was not related to changes in total Fyn protein expression levels (Fig. 2*A*, Fig. S1*A*). To assess whether PP2A-mediated alterations in detergent-insoluble Fyn levels are associated with modulation of Fyn activity state, cell fractions were also probed with validated antibodies recognizing SFKs phosphorylated (pSFK) at the conserved regulatory Tyr^416^ (numbering depending on species), a readout of SFK activity (1). Basal levels of active pSFK were observed in control N2a cells cultured in a “normal” serum-containing medium (Fig. 2*A*). Relative to controls, there was a marked increase in the pSFK signal in insoluble fractions that closely mirrored the increase in insoluble Fyn protein expression levels in WT- and LCMT1-expressing cells. Conversely, a similar reduction in the levels of pSFK and Fyn was observed in insoluble fractions from L309Δ- and PME1-expressing N2a cells. Yet, after normalizing the pSFK signal (apportioned to Fyn) for Fyn protein expression levels, we found no changes in the net phosphorylation of Fyn in any of the cell lines examined, relative to controls (Fig. S1*B*). Thus, under our experimental conditions, PP2A methylation influenced steady-state levels of active Fyn in detergent-insoluble fractions *via* mechanisms that do not implicate overall changes in Fyn activity or protein turnover.

Because neuronal Fyn is primarily concentrated in lipid rafts that resist extraction by nonionic detergents (9, 10, 11), perturbing PP2A methylation could more specifically alter the targeting of Fyn to these membrane microdomains. In this context, we have previously shown that pools of LCMT1 and methylated PP2Ac and PP2A/Bα holoenzymes are concentrated in lipid rafts, whereas demethylated PP2Ac is preferentially distributed in nonraft membrane microdomains purified from N2a cells (33). These observations suggest that Fyn and methylated PP2A/Bα enzymes are present in the same lipid raft compartment in N2a cells. To confirm this hypothesis, we reanalyzed fully characterized lipid raft and nonraft fractions obtained after membrane fractionation of N2a cells in an earlier study (33). Indeed, Fyn copurified with endogenous PP2Ac and Bα subunits in these flotillin-1–positive membrane rafts isolated from N2a cells (Fig. 2*E*). As expected, membrane cholesterol depletion by methyl-β-cyclodextrin (MβCD) induced the loss of both PP2A (33) and Fyn from these fractions (Fig. 2*F*). This effect was reversed by subsequent cholesterol replenishment using a preformed cholesterol–MβCD complex, demonstrating the cholesterol-dependent microdomain association of PP2A and Fyn. We next assessed how disrupting the integrity of PP2A methylation affects the levels of Fyn in N2a cell membrane rafts. We have previously reported that raft-bound PP2Ac levels are increased by ∼30% in WT-expressing N2a cells, compared with controls (33). We observed a similar pattern for Fyn. Expression of the WT enhanced the relative levels of raft-associated Fyn (Fig. 2, *G* and *H*), in agreement with the increase in Fyn amounts found in detergent-insoluble cell fractions (Fig. 2, *A* and *B*). In contrast, there was a marked reduction in raft-associated Fyn levels after expression of the methylation-incompetent L309Δ mutant. Unlike its WT counterpart, the L309Δ mutant is excluded from rafts, and raft-associated pools of PP2A become downregulated in L309Δ-expressing N2a cells (33). The decrease in raft-associated Fyn was also reminiscent of the loss of Fyn in detergent-insoluble fractions from L309Δ-expressing cells (Fig. 2, *A* and *B*). These findings indicate that altering PP2A methylation can negatively influence the targeting of Fyn to membrane rafts in N2a cells. The parallel loss of raft-associated PP2A (33) and Fyn (Fig. 2*G*) in L309Δ-expressing cells likely point to close spatial and functional interrelationships between the phosphatase and the kinase.

### Changes in PP2A methylation induce profound concomitant changes in the distribution of Fyn and F-actin in N2a cells

**Figure 3:**
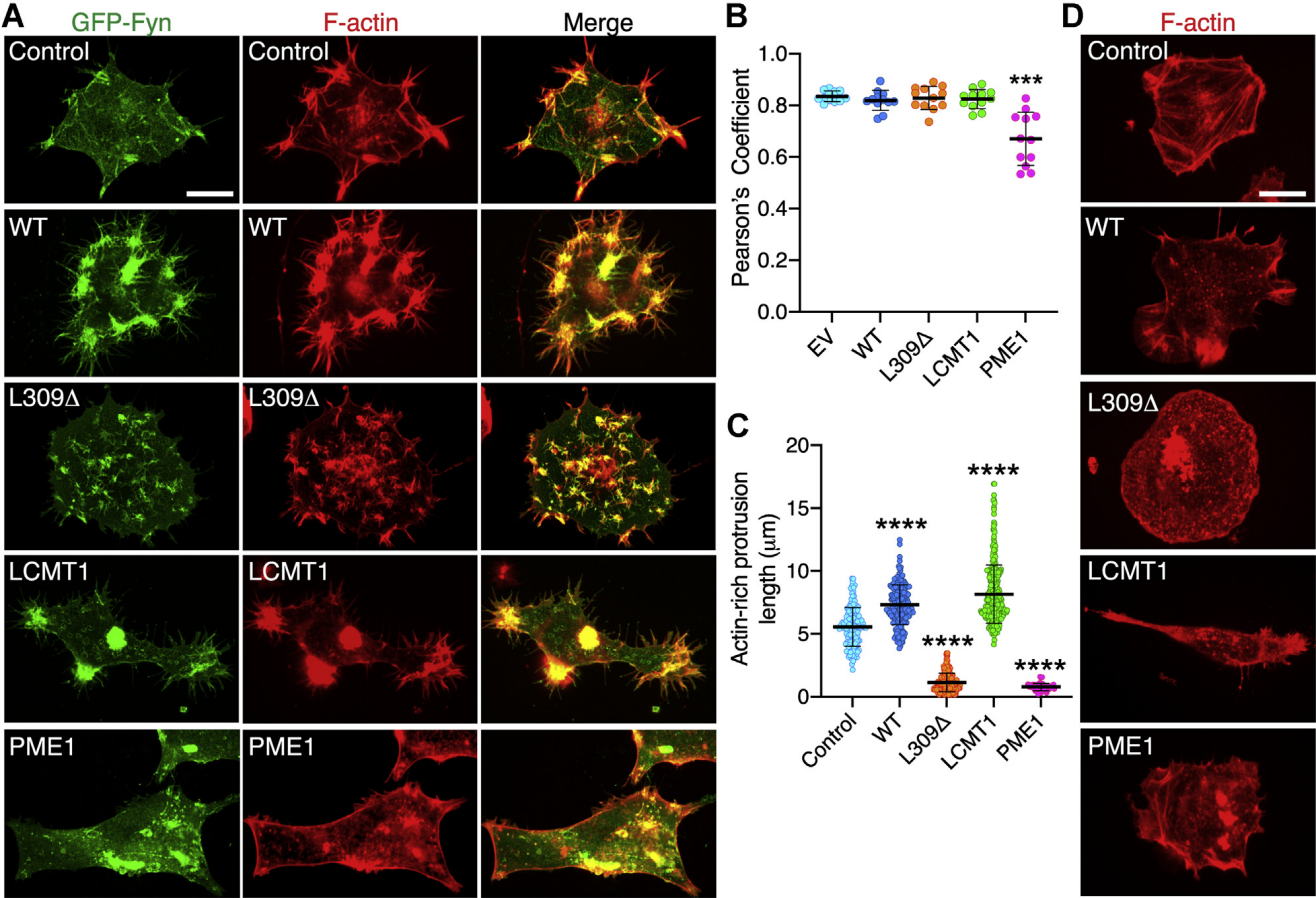
**Altering PP2A methylation induces defects in Fyn localization and F-actin organization in N2a cells.***A*, representative confocal images of the distribution of GFP–Fyn and F-actin in EV (control), WT-, L309Δ-, LCMT1-, or PME1-expressing N2a cells cotransfected with GFP–Fyn. *B*, Pearson’s correlation coefficients (mean ± SD, *n* = 12 cells/transfection from three separate experiments) showing colocalization of Fyn and F-actin in these cells. Data were analyzed using one-way ANOVA (F (4, 55) = 18.62, *p* < 0.0001) with post hoc Dunnett’s test; ∗∗∗*p* < 0.001, *versus* EV. *C*, cells were also analyzed for the length of actin-positive protrusions. Data (mean ± SD) were appraised using one-way ANOVA (F (4, 928) = 785; *p* < 0.0001) with post hoc Dunnett’s test. ∗∗∗∗*p* < 0.0001, *versus* control. *D*, F-actin distribution in the indicated N2a cell lines in the absence of GFP–Fyn. Images in panels *A* and *D* are representative of three separate experiments. Scale bars, 5 μm. EV, empty vector; N2a, Neuro-2a; LCMT1, leucine carboxyl methyltransferase 1; PP2A, protein phosphatase 2A.

To complement our biochemical approach, we compared by confocal microscopy the distribution of expressed GFP–Fyn in our N2a cell models. Cells were stained in parallel with phalloidin to reveal the organization of the F-actin cytoskeleton and cell shape. Earlier studies have established that Fyn is primarily localized at the cell plasma membrane, along actin-rich peripheral structures corresponding to focal adhesions, filopodia, or ruffles, depending on cell stimuli. Being subjected to important endocytic trafficking, small pools of the kinase can also be detected in perinuclear endosomes (7). Upon plating, neuronal cells also typically develop actin filopodial structures that grow into immature neurites during early stages of the differentiation process (35). In agreement with these studies, we observed that the bulk of GFP–Fyn was distributed along short actin-rich filopodia in control N2a cells (Fig. 3*A*). A similar colocalization of GFP–Fyn and F-actin was found in WT- and LCMT1-expressing N2a cells (Fig. 3, *A* and *B*). However, relative to controls, these cells displayed longer filopodia (Fig. 3*C*) where GFP–Fyn and F-actin coclustered. In addition, actin aggregates, which are associated with neurite initiation (35), were often present in these cells. In contrast, the distribution patterns of GFP–Fyn and F-actin were greatly disturbed in L309Δ- and PME1-expressing N2a cells. More central, diffuse, and disorganized GFP–Fyn staining alongside very short actin filamentous spikes were observed in N2a cells stably expressing the L309Δ mutant (Fig. 3, *A*–*C*). In PME1-expressing N2a cells, GFP–Fyn and F-actin colocalized in diffuse cytoplasmic patches rather than filopodia. GFP–Fyn was also absent from the cell periphery showing labeling of a cortical actin ring.

Thus, our findings uncover a concurrent reorganization of GFP–Fyn and the F-actin cytoskeleton after alterations in PP2A methylation. Because intact F-actin is critically required for proper peripheral membrane targeting of SFKs (36), we hypothesized that upsetting PP2A methylation homeostasis can influence the subcellular distribution of Fyn, at least in part by inducing a remodeling of the actin cytoskeleton. In support of this hypothesis, alterations in PP2A methylation state in our stable cell models were also associated with a profound rearrangement of the F-actin cytoskeleton and cell shape changes, in the absence of expressed GFP–Fyn (Fig. 3*D*).

### The interplay between PP2A methylation and Fyn regulates process outgrowth in N2a cells

**Figure 4:**
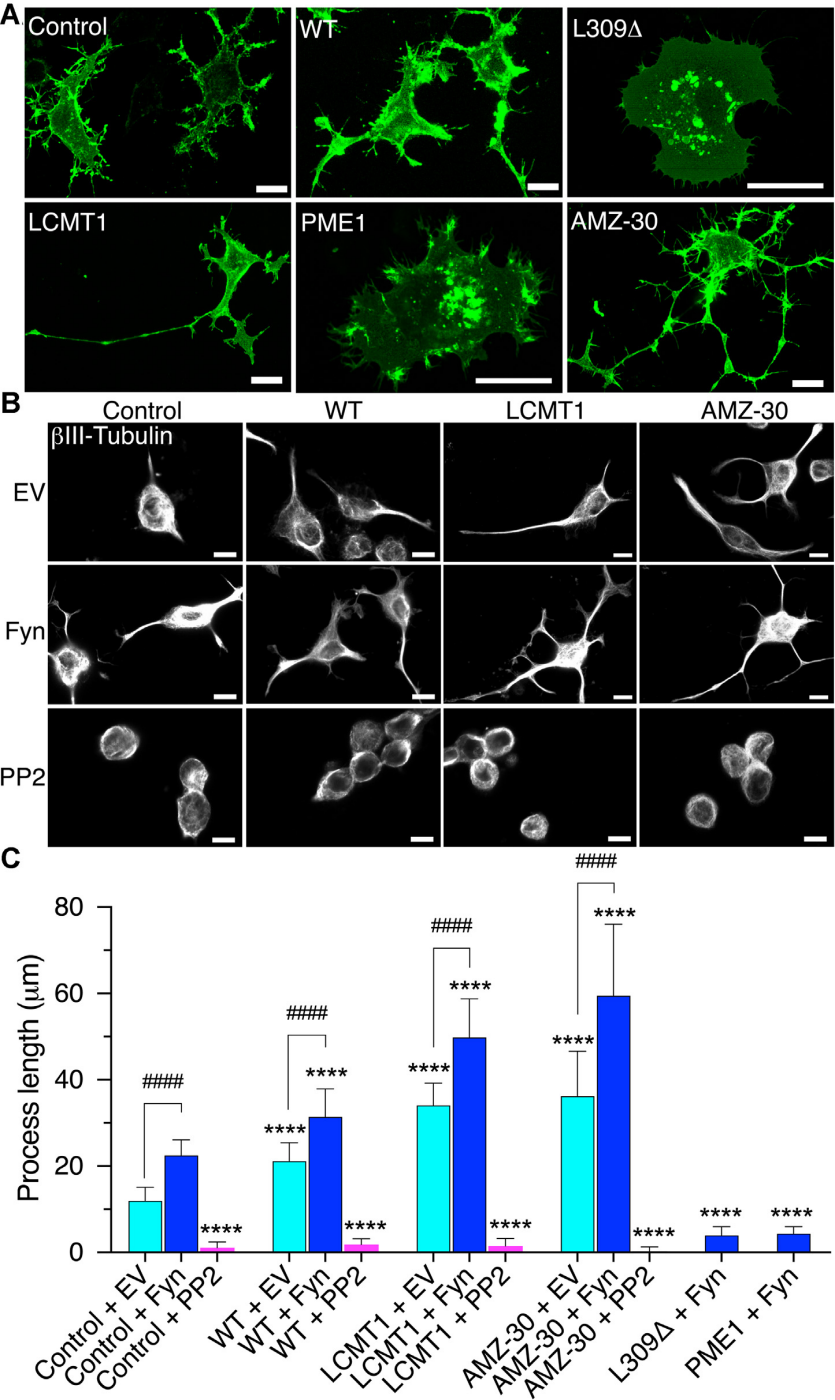
**PP2A methylation state affects Fyn-dependent process outgrowth.***A*, distribution of GFP–Fyn in control or WT-, L309Δ-, LCMT1-, or PME1-expressing N2a cells cotransfected with GFP–Fyn. Cells were incubated for ∼18 h in a low-serum medium to initiate differentiation before fixation. A subset of control cells was incubated in differentiation medium containing 1-μM AMZ-30. *B*, βIII-tubulin staining was used to assess neurite outgrowth in control or WT- or LCMT1-expressing N2a cells that were transfected with either EV or GFP–Fyn and incubated for ∼18 h in a low-serum medium in the absence or presence of PP2. Subsets of control cells were also incubated in the differentiation medium containing 1-μM AMZ-30. Confocal images shown in panels *A*–*B* are representative of three separate experiments. Scale bars, 10 μm. *C*, cells were comparatively analyzed for neuritic process length. Data are mean ± SD from *n* = 3 separate experiments and were appraised using two-way ANOVA (effect of PP2A methylation: F = 333.5, *p* < 0.0001; effect of Fyn–PP2: F = 2506, *p* < 0.0001; interaction: F = 113.8, *p* < 0.0001) with Tukey’s post hoc multiple comparisons test. ∗∗∗∗*p* < 0.0001, *versus* control; ^####^*p* < 0.0001. N2a, Neuro-2a; PP2A, protein phosphatase 2A.

Actin dynamics and reorganization are critical for neuritogenesis (35). Furthermore, one important role of Fyn clustering and activation in membrane rafts is to stimulate neurite outgrowth (13, 14, 15). In light of the effects of compromised PP2A methylation on the distribution of Fyn (Fig. 2) and F-actin (Fig. 3), we further investigated how changes in PP2A methylation affect Fyn-dependent process outgrowth. To that end, our N2a cell models were transfected with either GFP–Fyn or EV and incubated for ∼18 h in a low-serum medium to induce differentiation (37). Cells were comparatively analyzed for process outgrowth by assessing the distribution of transfected GFP–Fyn (Fig. 4*A*) or labeling neurites with the specific marker, βIII-tubulin (Fig. 4*B*). As expected, expression of GFP–Fyn in control N2a cells correlated with the outgrowth of highly branched Fyn-positive processes (Fig 4*A*), relative to EV-transfected N2a cells, which grew shorter neurites under the same differentiation conditions (Fig. 4, *B* and *C*). The stimulatory effects of Fyn on neuritogenesis were further accentuated in WT-transfected N2a cells. Cells coexpressing GFP–Fyn and LCMT1 displayed highly elongated processes. Likewise, incubation of GFP–Fyn–expressing N2a cells with AMZ-30, a specific PME1 inhibitor that blocks PP2A demethylation and enhances cellular levels of methylated PP2A (38), induced the formation of well-developed arrays of extended neurites. In contrast, expression of GFP–Fyn failed to induce process outgrowth in L309Δ- or PME1-expressing N2a cells (Fig. 4, *A* and *C*), which retained their characteristic rounded morphological appearance (see Fig. 3*A*). In these cell populations, GFP–Fyn was either distributed on short filamentous spikes as observed in undifferentiated cells (Fig. 3*A*) or retained in cytoplasmic and perinuclear vesicular patches. L309Δ- or PME1-transfected N2a cells also failed to differentiate in the absence of GFP–Fyn (37). Although we have previously reported that expression of WT and LCMT1 in N2a cells stimulates process outgrowth (37), expression of GFP–Fyn clearly promoted the growth of longer neurites in these cells, and in AMZ-30–treated N2a cells (Fig. 4, *B* and *C*). Conversely, neuritogenesis was completely abolished when cells were incubated in differentiation medium containing the Fyn inhibitor, PP2. These results indicate that intact PP2A methylation is essential for Fyn-dependent neuritogenesis while Fyn is required for PP2A methylation-dependent differentiation in N2a cells.

### PP2A methylation state affects the partitioning of APP in membrane microdomains and Fyn-dependent APP processing

**Figure 5:**
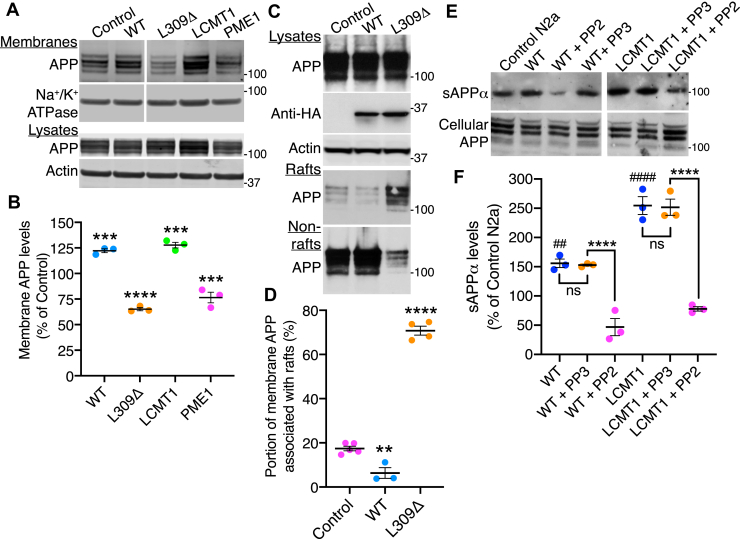
**PP2A methylation state affects APP membrane distribution and Fyn-dependent APP processing in N2a cells.***A*, representative immunoblots of endogenous APP distribution in purified membrane fractions from control or WT-, L309Δ-, LCMT1-, or PME1-expressing N2a cells. Quantitative analyses of the immunoblots from *n* = 4 separate experiments confirmed that there were no statistically significant changes in APP expression levels (*p* > 0.05) in total lysates from these cells. *B*, relative levels of membrane-associated APP in these cells. Data are mean ± SEM from *n* = 3 separate purifications and were analyzed using one-way ANOVA (F (4, 10) = 99.2, *p* < 0.0001) with post hoc Dunnett’s test. ∗∗∗*p* < 0.001, ∗∗∗∗*p* < 0.0001, *versus* control. *C*, representative immunoblots of APP distribution in aliquots of the same purified N2a cell raft and nonraft fractions analyzed in Figure 2*E*. Levels of expressed HA-tagged proteins and APP in corresponding total cell lysates are shown for reference. *D*, relative levels of raft-associated APP were quantified in EV-, WT-, and L309Δ-expressing N2a cells and are expressed as the percent of total membrane-associated APP. Data (mean ± SEM from *n* = 3–5 separate purifications) were appraised using one-way ANOVA (F (2, 9) = 382.5, *p* < 0.0001) with post hoc Dunnett’s test. ∗∗*p* < 0.01, ∗∗∗∗*p* < 0.0001, *versus* control. *E*, levels of secreted sAPPα species and corresponding cellular APP levels were comparatively analyzed by Western blot in WT- and LCMT1-expressing N2a cells, relative to control N2a cells, after incubation for 4 h in a conditioned media in the absence or presence of 5-μM PP2 or PP3. *F*, the release of sAPPα was quantified in these cells after normalization for total cellular APP levels. Data shown are the mean ± SEM from 3 separate assays and were analyzed using two-way ANOVA (cell line: F = 73.57, *p* < 0.0001; treatment: F = 112.7, *p* < 0.0001; interaction: F = 6.46, *p* = 0.01) using Tukey’s post hoc multiple comparisons test. ^##^*p* < 0.01, ^####^*p* < 0.0001, *versus* control N2a; ∗∗∗∗*p* < 0.0001; ns, not significant. APP, amyloid precursor protein; HA, hemagglutinin; LCMT1, leucine carboxyl methyltransferase 1; N2a, Neuro-2a; PP2Ac, catalytic “C” subunit of PP2A.

Besides regulating process outgrowth, Fyn critically regulates APP trafficking (5) and compartmentalization in membrane microdomains (11, 39). We thus hypothesized that, by affecting Fyn levels and distribution in membrane microdomains (Fig. 2), manipulating PP2A methylation could also alter the membrane distribution of APP in our N2a cell models. We first observed that, relative to controls, expression of WT and LCMT1 increased, whereas expression of L309Δ and PME1 decreased the levels of endogenous APP in plasma membrane fractions purified from N2a cells (Fig. 5, *A* and *B*). Quantification of Western blot analyses of total homogenates from these stable N2a cell lines confirmed that altering PP2A methylation state did not induce statistically significant changes in endogenous APP expression levels (Fig. 5*B*), as reported previously (23). These findings prompted us to further assess whether altering PP2A methylation promotes APP membrane microdomain switching in our models. To that end, we analyzed endogenous APP expression levels in aliquots of the same purified raft and nonraft preparations probed earlier for the presence of Fyn and PP2A (Fig. 2*E*). In contrast to Fyn (Fig. 2*E*), the bulk of endogenous APP partitioned in nonraft membrane fractions prepared from control N2a cells (Fig. 5, *C* and *D*), in agreement with earlier studies performed in neuronal tissue and cells, including N2a cells (16, 17, 18). Relative to controls, WT expression increased the levels of APP copurifying with nonraft membrane fractions. In contrast, the methylation-incompetent L309Δ mutant enhanced the relative proportion of APP segregating in rafts *versus* nonrafts. Thus, disrupting the integrity of PP2A methylation in N2a cells leads to increased partitioning of APP in membrane rafts.

Changes in the compartmentalization of APP in membrane microdomains critically affect APP processing (16, 17, 18, 19). Indeed, L309Δ-mediated redistribution of APP in rafts (Fig. 5, *C* and *D*) correlates with enhanced amyloidogenic processing of APP in L309Δ-expressing N2a cells (23). Conversely, we have previously shown that enhanced PP2A methylation in N2a cells stimulates the nonamyloidogenic processing of APP (23). Because the α-secretase cleavage of APP is also regulated by Fyn (39), we further examined whether Fyn inhibition can affect PP2A-mediated sAPPα secretion. Expression of either WT or LCMT1 in N2a cells boosted the release of sAPPα species (Fig. 5, *E* and *F*), in agreement with our earlier studies (23). Notably, these stimulatory effects were inhibited when cells were incubated in the presence of the PP2 inhibitor, but not PP3 (drug control). These findings suggest that PP2A methylation–dependent APP cleavage is dependent on Fyn.

### Disturbances in one-carbon metabolism that downregulate PP2A methylation alter the distribution of Fyn and Fyn-dependent process outgrowth in N2a cells

**Figure 6:**
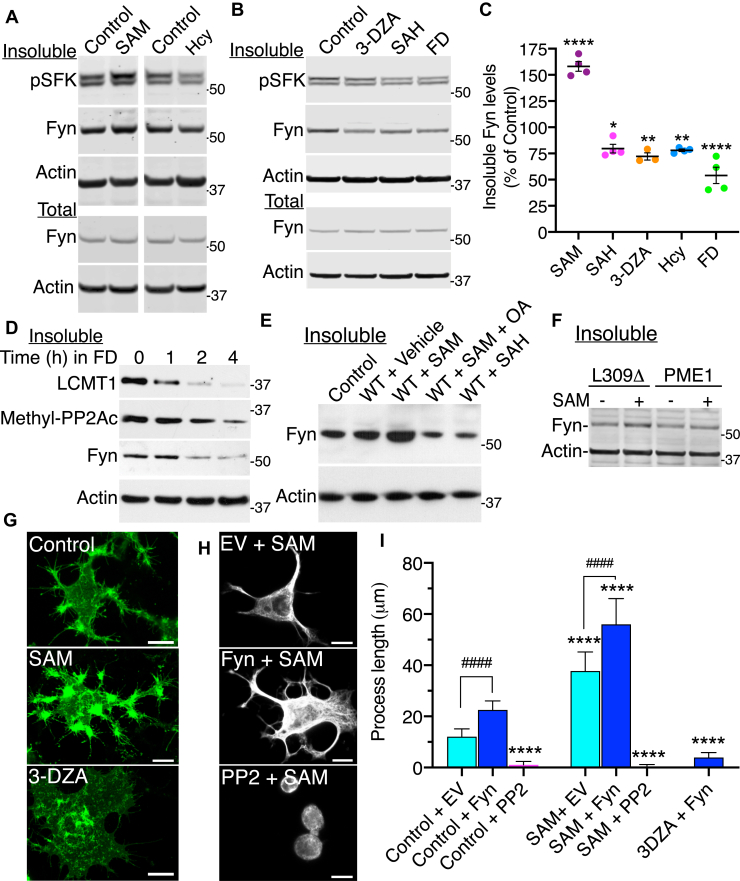
**Fyn distribution and Fyn-dependent process outgrowth are dependent on one-carbon metabolism in N2a cells.***A*, immunoblot analysis of Fyn and pY416-SFK (pSFK) in total lysates (total) and detergent-insoluble (insoluble) fractions from N2a cells that were incubated for ∼16 h with 100-μM SAM, 100-μM Hcy or vehicle (control). *B*, representative immunoblots of Fyn and pY416–SFK (pSFK) in total lysates and detergent-insoluble fractions from N2a cells that were incubated for ∼16 h with 50-μM 3-deazaadenosine (3-DZA), 100-μM SAH, or vehicle (control). A subset of cells was incubated for 4 h in a folate-deficient (FD) medium. Quantitative analyses of the immunoblots from *n* = 3 to 4 separate experiments revealed that incubation with either SAM, Hcy, SAH, 3-DZA, or FD did not induce any statistically significant changes (*p* > 0.05) in total Fyn protein expression levels, or total or detergent-insoluble Fyn phosphorylation levels, relative to vehicle-treated N2a cells. *C*, detergent-insoluble Fyn levels were quantified in these cells. Data shown are mean ± SEM from *n* = 3 to 4 independent experiments and were analyzed using one-way ANOVA (F (5, 17) = 69.37, *p* < 0.0001) with post hoc Dunnett’s test. ∗*p* < 0.05, ∗∗*p* < 0.01, ∗∗∗∗*p* < 0.0001, *versus* control. *D*, time-dependent changes in detergent-insoluble levels of Fyn, methylated PP2Ac, and LCMT1 enzymes in N2a cells switched for the indicated time from normal folate-containing medium to FD medium. *E*, immunoblot analysis of detergent-insoluble Fyn levels in control or WT-expressing N2a cells incubated for 16 h with 100-μM SAM, 100-μM SAH, or a combination of 100-μM SAM and 5-nM okadaic acid (OA). *F*, immunoblot analysis of detergent-insoluble Fyn and actin levels in L309Δ- and PME1-expressing N2a cells incubated for ∼16 h with 100-μM SAM or vehicle. For panels *D*–*F*, similar results were observed in three separate experiments. *G*, representative confocal images of GFP–Fyn in transfected N2a cells that were incubated for ∼18 h in a low-serum medium in the presence of 100-μM SAM, 50-μM 3-DZA, or vehicle (control) before fixation. Scale bars, 10 μm. *H*, N2a cells transfected with either EV or GFP–Fyn were incubated for ∼18 h in the differentiation medium in the absence or presence of 5-μM PP2 and labeled with anti-βIII-tubulin antibodies. Scale bars, 10 μm. *I*, quantification of the neurite length in these cells. Data shown are mean ± SD from cells from 3 separate experiments and were analyzed with two-way ANOVA (effect of SAM: F = 1152, *p* < 0.0001; effect of Fyn–PP2: F =1600, *p* < 0.0001; interaction: F = 342.1, *p* < 0.0001) with post hoc Tukey’s test. ∗∗∗∗*p* < 0.0001, *versus* control + EV or control + Fyn; ^####^*p* < 0.0001. EV, empty vector; FD, folate-deficient; Hcy, homocysteine; LCMT1, leucine carboxyl methyltransferase 1; N2a, Neuro-2a; PP2Ac, catalytic “C” subunit of PP2A.

All cellular methylation reactions depend on the availability of the universal methyl donor, SAM, whose levels are tightly regulated by one-carbon metabolism (40). We have previously reported that alterations in one-carbon metabolism inhibit LCMT1-dependent PP2Ac methylation in N2a cells and *in vivo*, thereby affecting PP2A subunit composition and substrate specificity (23, 34, 41). To further explore the PP2A methylation/Fyn connection, we investigated the effects of manipulating one-carbon metabolism on Fyn regulation. N2a cells were first incubated in an SAM-enriched medium, which boosts LCMT1-mediated PP2Ac methylation (23) and increases amounts of methylated PP2A enzymes targeted to the plasma membrane (33). Incubation with SAM enhanced the basal levels of endogenous Fyn in detergent-insoluble cell fractions, relative to vehicle-treated controls (Fig. 6, *A* and *C*). Next, N2a cells were subjected to various treatments known to deregulate one-carbon metabolism and interfere with the normal methylation cycle. Cells were incubated with Hcy, SAH, or a SAH hydrolase inhibitor (3-deazaadenosine [3-DZA]); subsets of cells were also switched to a folate-deficient (FD) medium to induce short-term folate deficiency (Fig. 6, *A* and *B*). Albeit their mechanisms of action are distinct, these treatments culminate to elevate intracellular Hcy and alter the cellular SAM/SAH ratio that controls cellular methylation potential (40). In turn, decreased SAM availability and/or increased cellular levels of SAH, a potent inhibitor of methyltransferases, induce the inhibition of LCMT1-dependent PP2A methylation and loss of membrane-associated methylated PP2A enzymes in N2a cells (23, 33, 34). Likewise, these treatments led to a pronounced reduction in detergent-insoluble Fyn levels (Fig. 6, *A*–*C*). As observed when deregulating PP2A methylation (Fig. 2*A*), manipulations of one-carbon metabolism induced parallel changes in pSFK immunoreactivity and Fyn expression levels in detergent-insoluble cell fractions (Fig. 6, *A*–*C*). After normalization, there were no statistically significant changes in either net Fyn activity or total Fyn expression levels in untreated or treated N2a cells.

Notably, incubation of N2a cells in the FD medium caused a time-dependent parallel loss of LCMT1, methylated PP2A, and Fyn enzymes from detergent-insoluble fractions (Fig. 6*D*). Moreover, treatment of N2a cells with SAM further synergized with WT expression to enhance insoluble Fyn levels, whereas SAH blocked the ability of WT to increase insoluble Fyn levels (Fig. 6*E*). SAM-mediated increase in insoluble Fyn levels was also abolished when cells were coincubated with the PP2A inhibitor, okadaic acid (OA). However, incubation with SAM was unable to rescue the loss of insoluble Fyn levels induced by L309Δ or PME1 expression in N2a cells (Fig. 6*F*). Together, these findings indicate that the status of one-carbon metabolism can concurrently influence the membrane distribution of PP2A and Fyn. They support the hypothesis that metabolic-induced deregulation of PP2A methylation is a major contributor to Fyn deregulation. In this context, it has been reported in non-neuronal COS-1 cells that Fyn is trimethylated at Lys^7/9^ (42). This observation raised the possibility that altering one-carbon metabolism could also impact Fyn regulation in a PP2A-independent manner, by directly affecting its methylation state. To address this hypothesis, GFP–Fyn immunoprecipitates were prepared from transfected N2a cells treated with SAM (to enhance methylation), 3-DZA (to inhibit methylation), or vehicle and then analyzed by Western blot with validated anti-methyl-lysine antibodies (Fig. S2). Although methyl-sensitive proteins were clearly present in the GFP–Fyn immunoprecipitates, we were unable to detect any immunoreactivity of GFP–Fyn with these antibodies under our experimental conditions.

We next investigated whether Fyn-dependent process outgrowth was susceptible to changes in the SAM/SAH ratio. Relative to vehicle-treated controls, incubation with SAM enhanced GFP–Fyn–mediated differentiation of N2a cells (Fig. 6, G–I). This stimulating effect and the pattern of GFP–Fyn distribution in SAM-treated cells were highly reminiscent of those induced by AMZ-30, WT, and LCMT1 (Figs. 3 and 4). Notably, SAM-mediated N2a cell differentiation was abolished by PP2. Fyn-dependent process outgrowth was also inhibited by 3-DZA. The distribution of GFP–Fyn in 3-DZA–treated cells was strikingly similar to that observed in L309Δ-expressing cells (Figs. 3*A* and 4*A*). Thus, the integrity of one-carbon metabolism is essential for Fyn-dependent process formation in N2a cells.

### Elevated Hcy levels influence the amounts of methylated PP2A and Fyn enzymes in detergent-insoluble fractions prepared from acute mouse brain slices

**Figure 7:**
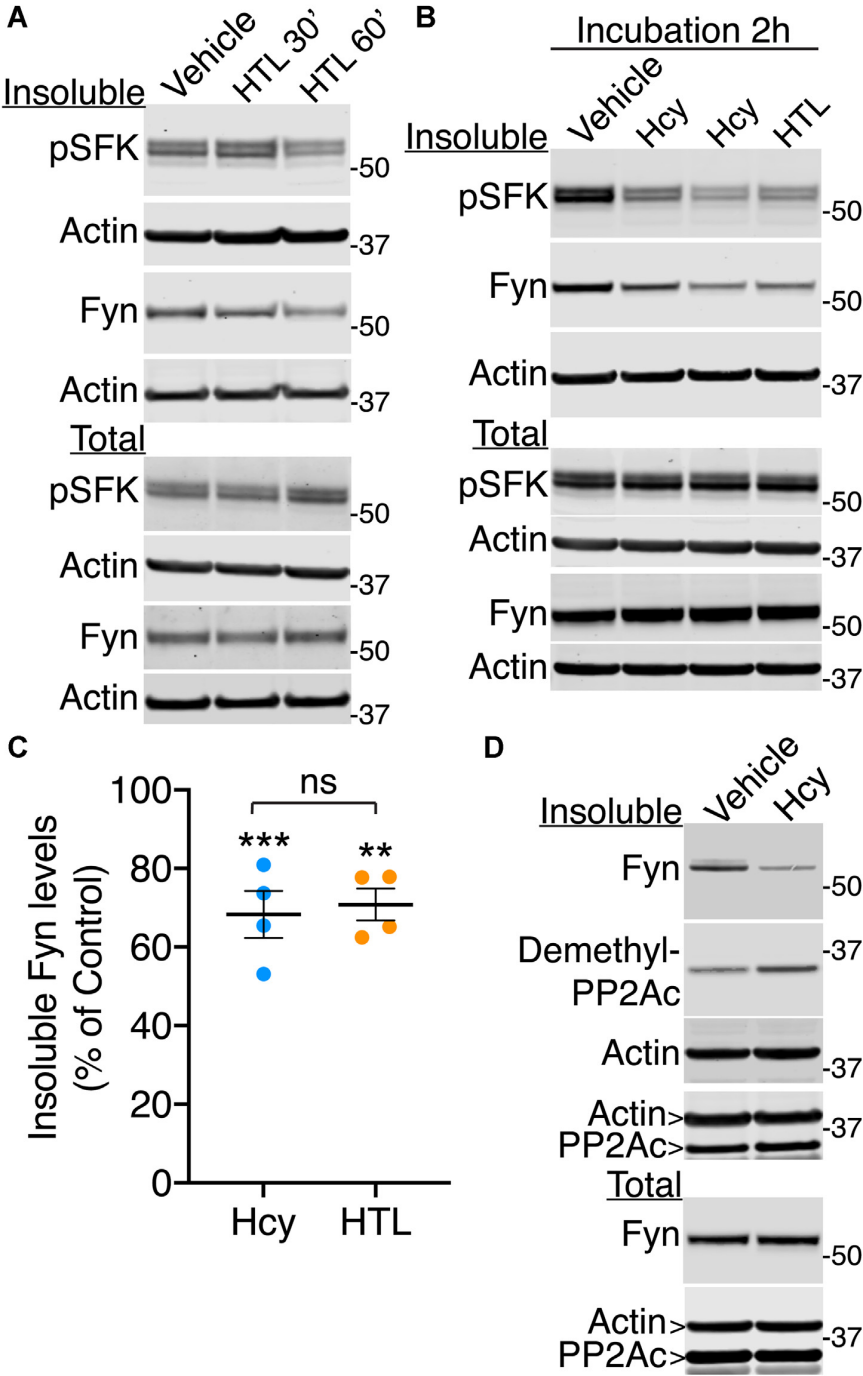
**Elevated levels of Hcy or its thiolactone derivative induce concomitant PP2A demethylation and alterations in Fyn distribution in acute mouse brain slices.***A*, representative immunoblots of Fyn and pSFK in total extracts (total) and detergent-insoluble (insoluble) fractions prepared from acute mouse cortical slices incubated for 30 or 60 min with 200-μM Hcy-thiolactone (HTL). *B*, representative immunoblots of pSFK and Fyn in acute brain slices incubated for 2 h with either 200-μM Hcy or HTL. Two separate slices treated with Hcy are shown. Quantitative analyses of the immunoblots from *n* = 4 separate experiments revealed that total Fyn protein expression levels, or total or detergent-insoluble Fyn phosphorylation levels were not statistically significant different (*p* > 0.05) in Hcy or HTL-treated, relative to, vehicle-treated slices. *C*, quantification of detergent-insoluble Fyn levels in Hcy- or HTL-treated slices, expressed as percent of vehicle-treated, control slices. Data shown are mean ± SEM from 4 separate mouse brain tissue and were analyzed using one-way ANOVA (F (2, 9) = 17.88, *p* = 0.0007) with post hoc Dunnett’s test; ∗∗*p* <0.01, ∗∗∗*p* <0.001, relative to vehicle-treated slices. *D*, comparative immunoblot analysis of demethylated PP2Ac, Fyn, and actin in total extracts and detergent-insoluble fractions prepared from acute mouse cortical slices incubated for 2 h with 200-μM Hcy. Duplicate blots were probed for total PP2Ac levels and actin. Quantitative analyses of the immunoblots showed a ∼58 ± 9% increase (*n* = 3 separate experiments; *p* < 0.0001; student *t*-test) in demethylated PP2A levels in detergent-insoluble fractions from Hcy-, relative to, vehicle-treated slices. Hcy, homocysteine; HTL, Hcy thiolactone; PP2Ac, catalytic “C” subunit of PP2A; pSFK, phosphorylated SFK.

To validate the studies performed in N2a cells, we examined the effects of disturbing Hcy metabolism on the detergent insolubility of Fyn in acute mouse brain slices. Fyn-enriched detergent-insoluble fractions were prepared from cortical slices that had been incubated for up to 2 h in the presence of Hcy or its metabolite, Hcy thiolactone (HTL) (Fig. 7, *A*–*C*). This short treatment was preferentially chosen to prevent putative protein loss due to ultimate Hcy neurotoxicity. Relative to vehicle-treated slices, incubation with either Hcy or HTL induced a time dependent loss of detergent-insoluble Fyn levels, with a mean ∼30% loss found at the 2-h time point. As observed in N2a cells (Figs. 2 and 6), the loss of Fyn was accompanied by a similar and parallel decrease in the signal for pY416–Fyn in detergent-insoluble fractions. Indeed, after normalization, there were no significant changes in net insoluble Fyn activity in Hcy- or HTL-treated compared with vehicle-treated slices. The loss of Fyn also coincided with a large increase in demethylated PP2A in detergent-insoluble fractions from Hcy-treated slices, relative to vehicle-treated slices (Fig. 7*D*). These results indicate that the abnormal elevation of Hcy can induce a concomitant demethylation of PP2A and redistribution of active Fyn enzymes.

## Discussion

Methylation is a key regulatory post-translational mechanism that controls biogenesis of PP2A/Bα holoenzymes and PP2A subunit composition, thereby influencing PP2A targeting, substrate specificity, and interactions with numerous proteins and regulators (22). Here, using N2a cells, we first show that manipulating PP2A methylation affects the distribution and function of Fyn, a major signaling enzyme deregulated in AD and cancer. The subcellular distribution of Fyn is regulated by trafficking to and internalization from the plasma membrane; owing to N-terminal lipid modifications, Fyn is preferentially targeted to membrane rafts, regardless of its activity (7, 8, 43, 44, 45). We found that enhanced cellular PP2A methylation was associated with increased levels of Fyn in detergent-insoluble N2a cell fractions and membrane rafts and enhanced clustering of Fyn along peripheral actin-rich filopodia. Conversely, the accumulation of demethylated PP2A promoted a steady-state loss of membrane- and raft-associated Fyn and its mislocalization. These effects were not associated with changes in net Fyn activity or expression levels; rather, they closely correlated with a reorganization of the F-actin cytoskeleton. Although general PP2A activity has been implicated in the complex regulation of actin dynamics, underlying mechanisms and contribution of specific PP2A isoforms remain poorly characterized (46). Nevertheless, an intact actin cytoskeleton is critically required for proper peripheral membrane targeting of Fyn (36). Our findings suggest that altering PP2A methylation in N2a cells interferes with the normal distribution of Fyn, likely in part by deregulating F-actin dynamics. This hypothesis is further supported by the strong functional link between cytoskeletal reorganization and clustering of proteins in membrane rafts, which serve as signaling platforms regulating adhesion, differentiation, and polarity (47). These membrane microdomains are also involved in protein sorting, endocytosis, and recycling from/to the plasma membrane (48). Moreover, deregulation of PP2A promotes the endocytosis of E-cadherin by inducing F-actin disassembly (49). The pattern of Fyn distribution in L309Δ- and PME1-expressing N2a cells suggests that enhanced PP2A demethylation could similarly promote Fyn internalization *via* actin-dependent mechanisms. Yet, defects in Fyn trafficking could also occur by other mechanisms, based on the role of PP2A in dephosphorylating adaptor proteins regulating clathrin-mediated endocytosis (50).

We also observed that increasing PP2A methylation stimulated Fyn-dependent process outgrowth, whereas altering PP2A methylation abolished it. Because activation of raft-associated Fyn (13, 14, 15) and actin remodeling (35) are intimately linked with neuritogenesis, it is likely that PP2A influences Fyn-dependent N2a cell differentiation by affecting F-actin dynamics and Fyn targeting to rafts. For instance, the reorganization of cortical actin into aggregates and filopodia, which is required for neurite initiation (35), was prevalent in WT- and LCMT1- but absent in PME1-expressing N2a cells. However, additional direct and indirect mechanisms, such as PP2A-induced changes in microtubule stability (46) and Fyn-mediated F-actin dynamics (1), could also be involved.

Our data also indicate that deregulation of PP2A in N2a cells influences the clustering of APP in membrane microdomains, which governs APP processing (17, 18, 19). Accordingly, we found that enhanced association of APP with rafts (Fig. 5*D*) coincided with enhanced β-secretase cleavage of APP (23) in L309Δ-expressing N2a cells. Conversely, expression of the WT in N2a cells increased the relative levels of endogenous APP in nonraft plasma membrane microdomains and sAPPα secretion (Fig. 5). In agreement with decreased sAPPα release in Fyn KO mice (39), inhibiting Fyn abolished both WT- and LCMT1-mediated sAPPα secretion in N2a cells. These data further cement the existence of a close methylated PP2A/Fyn functional inter-relationship in regulating APP.

Phosphorylation-dependent protein–protein interactions also shape APP localization and processing (3, 25). Fyn-mediated Tyr phosphorylation regulates the association of APP with adaptor proteins and promotes the sorting of APP to lipid rafts (11). Aberrant Fyn activation in AD has been linked to enhanced Fyn–APP interactions, deficits in APP sorting and trafficking, and amyloidogenesis (5, 51). We found that PP2A/Bα holoenzymes copurified in membrane rafts and coimmunoprecipitated with detergent-insoluble Fyn. This indicates the existence of membrane-bound Fyn–PP2A complexes; however, these protein–protein interactions may be indirect because purified Fyn and PP2A/Bα holoenzymes do not associate *in vitro* (52). Relative to control N2a cells, expression of the WT enhanced Fyn levels in rafts while increasing the ratio of APP partitioning in nonraft membrane microdomains, suggesting that WT promotes the segregation of Fyn from its substrate, APP. In contrast to WT, the methylation-incompetent and B binding–incompetent L309Δ mutant failed to associate with Fyn and promoted the sorting of APP into rafts. Whereas the expression of L309Δ decreased total Fyn levels in rafts, enhanced targeting of APP to rafts may increase the potential for functional interactions of APP with the active kinase still present in these microdomains. Enhanced PP2A demethylation in N2a cells (23) also promotes phosphorylation of APP at Thr^668^, which impacts the APP interactome (25) and APP distribution (3). In neurons, pThr^668^–APP species are concentrated in endosomes, favoring APP β-secretase cleavage (3). Based on these findings, it is tempting to speculate that PP2A methylation regulates the formation of localized protein scaffolds that play a crucial role in directing the trafficking and processing of APP toward the competing amyloidogenic or nonamyloidogenic pathways. In this context, it is noteworthy that the L309Δ mutant fails to associate not only with Fyn (Fig. 1*B*) but also with tau proteins (23). Enhanced formation of Fyn–tau scaffolds plays a key role in mediating Aβ-induced synaptic dysfunction and excitotoxicity in AD (4). Because PP2A/Bα and Fyn compete for tau binding, disruption of normal PP2A–Fyn and PP2A–tau protein–protein interactions as a result of PP2A demethylation would enhance the potential for neurotoxic Fyn–tau interactions (53). Thus, interfering with homeostatic PP2A methylation has the potential, *via* several intricate mechanisms, to deregulate the function of key players in AD pathogenesis.

Using N2a cells and mouse brain slices, we also established a link between one-carbon metabolism and the regulation of Fyn distribution. Metabolic disturbances that lead to elevated Hcy levels and altered cellular methylation potential induced a concomitant loss of methylated PP2A and Fyn enzymes from detergent-insoluble fractions; conversely, boosting cellular methylation led to their coenrichment. In an earlier mass spectrometry study, Fyn was reported to be trimethylated on Lys residues within the SH4 domain; a regulatory link between methylation and Fyn targeting and function was further proposed based on the use of Lys mutants in COS-1 cells (42). These observations could provide a plausible mechanism by which altering one-carbon metabolism can directly affect Fyn methylation state and thereby affect its distribution. However, to the best of our knowledge, Fyn methylation has never been confirmed in any follow-up studies, and the identity of the Fyn methyltransferase remains unknown to date. Proper validation of protein Lys methylation is challenging, requiring several approaches to avoid pitfalls (54). Assigning effects of Lys mutants to changes in Fyn methylation (53) may be confounded by the fact that Fyn also undergoes acylation in the same N-terminal domain, which controls Fyn association with membrane rafts (44). Under our experimental conditions, we were unable to detect Fyn methylated on Lys residues, arguing against a prevalent direct role of Lys methylation in regulating Fyn distribution. Yet, we do not exclude the possibility that Fyn undergoes methylation on other yet unidentified amino acids, which could render Fyn directly susceptible to alterations in one-carbon metabolism. Nevertheless, our findings in N2a cells (Fig. 6, *E* and *F*) and brain slices (Fig. 7) strongly support the hypothesis that altered PP2A methylation actively contributes to deregulation of Fyn in response to disturbances in the methylation cycle. Yet, PP2A- and methylation-independent mechanisms, such as oxidative stress (26), could also participate in Fyn dysregulation in response to altered folate and Hcy metabolism.

The crosstalk between Hcy metabolism and the major signaling molecules, PP2A and Fyn, is of particular importance for the AD and cancer fields. Hyperhomocysteinemia is an established risk factor for AD (27) and is experimentally associated with the development of hallmark pathological features of AD, including tau and APP phosphorylation, and amyloidogenesis (26). Disturbed Hcy metabolism is strongly associated with cancer (28). Hyperhomocysteinemia promotes PP2A demethylation *in vivo* (23, 55). Alterations in PP2A methylation are found in patients with AD (21) and cancer (56). In our neuroblastoma model, they promote a loss of Fyn from membrane rafts. Because the confinement of Fyn in membrane rafts limits its ability to promote cell transformation (44, 57), it is possible that deregulation of Fyn contributes to the role of PP2A in cancer.

Collectively, our findings demonstrate that the integrity of one-carbon metabolism and PP2A methylation are essential for proper regulation of Fyn and APP. Our study identifies an important link between metabolic pathways and multifunctional signaling molecules currently being targeted for AD and cancer therapies.

## Experimental procedures

### Materials and reagents

Unless indicated, all chemicals and drugs were from Sigma-Aldrich/Merck Millipore. Primary antibodies used in this study included the following: Rabbit anti-HA clone C29F4, anti-pSrc Tyr416 clones 100F9 and D49G4, anti-Na^+^/K^+^ ATPase #3010, and anti-GFP clone D5.1 (Cell Signaling Technology); rabbit Anti-APP clone Y188 (Abcam); mouse anti-HA clone 16B12 (Covance); rabbit anti-actin #AAN01 (Cytoskeleton Inc); anti-transferrin receptor clone H68.4 (Thermo Fisher Scientific); rabbit anti-methylated Lysine (Enzo Life Sciences); mouse anti-Fyn clone 25 #610163, anti-flotillin-1 clone 18, and anti-PP2Ac_α_ clone 46 (BD Transduction Laboratories); rabbit anti-Fyn clone EPR5500, mouse anti-Bα clone 2G9, anti-actin clone C4, anti-LCMT1 clone 4A4, anti-APP clone 22C11 (MAB348), and anti-demethyl PP2Ac clone 1D6 (Merck Millipore).

### Cell culture and transfection

Mouse N2a neuroblastoma cells were obtained from the American Type Culture Collection. N2a cells stably expressing myc-tagged PME-1, HA-tagged LCMT1, HA-tagged WT PP2Ac, or the HA-tagged methylation site L309Δ C subunit mutant have been extensively characterized in previous studies (23, 31, 33, 34). Control and stable cell lines were maintained in Dulbecco’s modified Eagle’s medium (DMEM, Thermo Fisher Scientific) containing 2.5-mM Hepes, pH 7.4, 10% fetal bovine serum (FBS, Bovogen Biologicals), and 10-μg/ml gentamycin (Thermo Fisher Scientific). In some experiments, control and stable cells lines were transiently transfected with the indicated plasmids using METAFECTENE PRO reagent, following the manufacturer’s instructions (Biontex laboratories, Germany). Plasmids used in this study included the following: pAcCMV Fyn–GFP plasmid encoding GFP-tagged human Fyn (OriGene); peCFP-APP plasmid encoding human WT APP695 (gift from Dr Ottavio Arancio, Columbia University, New York, NY); and Bα/pcDNA5/TO plasmid encoding Flag-tagged PP2A Bα (PPP2R2A) subunit (58) (gift from Dr Brian Wadzinski, Vanderbilt University, Nashville, TN). All plasmids were verified by sequencing. Cells mock-transfected with EVs were used as “controls.” Partial knockdown of endogenous LCMT1 in N2a cells was performed using transient transfection with small interfering RNA (siLCMT1) shown to specifically target mouse LCMT1; cells transfected with mismatch siRNA (siRNA control) were used as controls. Experimental conditions were optimized to prevent cell death ultimately caused by the complete or prolonged loss of LCMT1 (33, 34).

### Cell treatment and differentiation

Unless otherwise indicated, all experiments and incubation with compounds were performed in ∼80% confluent cells cultured in a regular cell culture medium. To assess the role of one-carbon metabolism, cells were incubated for ∼16 h with 100-μM SAM, 100-μM SAM + 5-nM 100 OA, 100-μM SAH, 50-μM 3-DZA, 1 μM AMZ-30, or vehicle (33). Folate deficiency was induced by switching N2a cells cultured in normal folate-containing medium to folate-free RPMI-1640 medium supplemented with 2% dialyzed FBS (Thermo Fisher Scientific) (34). To assess the role of cholesterol, subsets of cells were incubated at 37 °C for 15 min in a serum-free medium with 1% MβCD premixed or not with 100 μg/ml cholesterol, before harvesting for membrane raft purification (33). To assess the role of Fyn, cells were incubated for the indicated time in the presence of 5-μM PP2 or PP3 (control drug for the SFK inhibitor, PP2). sAPPα secretion was analyzed 4 h after incubation of N2a cells in the conditioned media, exactly as described previously (23). To study neurite outgrowth, cells were plated in a regular medium 24 h after transfection onto poly-L-lysine–coated glass coverslips. Five hours after plating, cells were switched to DMEM containing 0.5% FBS in the absence or presence of the indicated drugs and incubated for ∼18 h before fixation.

### Confocal microscopy

To visualize F-actin, cells were fixed for 20 min with 4% paraformaldehyde, permeabilized for 5 min with PBS containing 0.1% Triton X-100, washed, and incubated for 1 h in PBS containing 3% bovine serum albumin. Cells were labeled with Alexa Fluor^594^ conjugated phalloidin to detect F-actin (Thermo Fisher Scientific). GFP–Fyn was directly visualized in fixed cells. To label neurites, N2a cell lines were fixed for 5 min at −20 °C with absolute methanol before staining with rabbit anti-βIII-tubulin antibodies (Abcam #18207) followed by Alexa Fluor^594^–conjugated secondary antibody (Thermo Fisher Scientific #A27034) (37). After washing in PBS, all samples were mounted with Fluoromount (ProSciTech) and examined on a Nikon Eclipse 80i confocal microscope using a 60x objective. Captured images (z-stacks) were exported to NIH ImageJ/Fiji for analyses of either the protrusion length or protein colocalization. Images were transferred to Adobe Photoshop/Illustrator 2020 (Adobe Systems Incorporated) for figure preparation.

### Cell lysis and subcellular fractionation

After washing with PBS, total N2a cell homogenates (100-mm dishes) were prepared in 400-μl buffer 1 [10-mM Tris, pH 7.4, 150-mM NaCl, 1-mM dithiothreitol, 0.5-μM OA, 5-mM PMSF, 1% NP-40, Sigma Protease Inhibitor Cocktail, and Sigma Phosphatase Inhibitor Cocktail] using a mortar and pestle. In some experiments, total cell lysates were further centrifuged for 90 min at 20,000*g* to generate NP-40 detergent–soluble (supernatant) and NP-40 detergent–insoluble (pellet) fractions. The detergent-insoluble cell pellet was resuspended in 200 μl of buffer 2 (buffer 1 + 0.5% sodium deoxycholate) and carefully homogenized for 80 s using a mortar and pestle. For immunoprecipitation assays, homogenized total and insoluble fractions were cleared by centrifugation at 13,000*g* for 3 min at 4 °C. For Western blot analyses, total homogenates and detergent-insoluble fractions were further sonicated before clarification. The protein concentration was determined in diluted aliquots of homogenates using the Bradford protein assay kit (Bio-Rad). Previously purified NP-40–insoluble fractions from N2a cells transiently transfected with validated siLCMT1 or siRNA control (33) were also reanalyzed here by immunoblotting. Purification of the plasma membrane from N2a cells was carried out by ultracentrifugation (33). Validated detergent-free procedures based on fractionation of the plasma membrane by centrifugation on an OptiPrep gradient were used to purify raft and nonraft membrane microdomains from N2a cells (33). Aliquots of the same N2a cell membrane microdomain preparations characterized in a previous study (33) were reanalyzed here by Western blot for the presence of Fyn and APP.

### Immunoprecipitation

Immunoprecipitates were prepared from total homogenates or detergent-insoluble fractions from N2a cells or mouse cortical tissue (∼500 μg proteins/assay). Immunoprecipitation of transfected proteins was performed by incubating samples overnight at 4 °C with either anti-Flag–coupled (clone M2, Sigma #M8823), anti-GFP–coupled (clone RQ2, MBL International #D153–9), or anti-HA–coupled (clone C29F4; Cell Signaling Technology #11846) magnetic beads. When immunoprecipitating endogenous proteins, homogenates were precleared for 1 h at 4 °C before overnight incubation with the indicated antibodies. Samples were then incubated for 1 h at 4 °C with PureProteome Protein A/G mix magnetic beads (Merck Millipore). Magnetic beads were washed 5 times in buffer 2 before being resuspended in a gel loading buffer. Input fractions (∼50 μg proteins) and corresponding immunoprecipitates were analyzed by Western blotting.

### Mouse brain tissue analyses

Brains were rapidly removed from 8- to 11-month-old female C57/BL6 mice that were sacrificed for another project approved by the Animal Care and Ethics Committee of the University of Newcastle. For the preparation of acute slices, brains were immediately immersed in ice-cold, oxygenated, sucrose-substituted artificial cerebrospinal fluid (250-mM sucrose, 25-mM NaHCO_3_, 10-mM glucose, 2.5-mM KCl, 1-mM NaH_2_PO_4_, 1-mM MgCl_2_, and 2.5-mM CaCl_2_) (59). Cortical coronal slices (∼400 μm thick) were obtained using a vibratome (Leica VT-1200S, Heidelberg, Germany) and transferred to an interface storage chamber containing oxygenated artificial cerebrospinal fluid (118-mM NaCl substituted for sucrose in sucrose-substituted artificial cerebrospinal fluid). Slices were allowed to recover for 1 h at 22 to 24 °C before incubation for the indicated time into RT oxygenated artificial cerebrospinal fluid containing the indicated compounds or vehicle. Slices were then harvested for further Western blot analyses. Total homogenates and NP-40 detergent-insoluble fractions were prepared from either acute slices or fresh mouse cortical tissue as described above for N2a cells.

### Gel electrophoresis and Western blotting

Protein samples (∼50-μg proteins/lane) were resolved on NuPAGE 4 to 12% Bis-Tris gels (Thermo Fisher Scientific). Prestained Protein Standards (Bio-Rad) were used as molecular weight markers. Membrane raft fractions were analyzed by immunoblotting using chemiluminescence as described previously (33). Other Western blot analyses were performed using the indicated primary antibodies, followed by Infrared IRDye-labeled secondary antibodies, and visualized using the Odyssey Infrared imaging system (LI-COR Biosciences). In most cases, blots were cut between molecular weight markers to allow simultaneous immunostaining and reprobing of the top and bottom parts with distinct antibody species. Band intensity was determined using the associated Image Studio Lite, version 5.0, Software (LI-COR Biosciences) to accurately quantify protein expression levels. Anti-actin antibodies were used to normalize for protein loading. Anti-pTyr^416^ SFK antibodies were used to assess the phosphorylation state of Fyn, which was determined after normalizing the pSFK signal corresponding to Fyn (determined as being the fastest migrating band) for total Fyn expression levels and protein loading. PP2A demethylation was assessed as described previously (33).

### Statistics

Data were analyzed for normal distribution and statistical significance using GraphPad Prism 9.

## Data availability

All the data supporting our conclusions are presented in this article. All materials are available upon request.

## Conflicts of interest

The authors declare that they have no conflicts of interest with the contents of this article.
