# Supplementary material for: Disturbances in PP2A methylation and one-carbon metabolism compromise Fyn distribution, neuritogenesis, and APP regulation
Source: J Biol Chem. 2021 Jan 7;296:100237. doi: 10.1074/jbc.RA120.016069 (PMC7948947; doi:10.1074/jbc.RA120.016069)
Supplement: Supplementary Figures [file mmc1.pdf]

SUPPORTING INFORMATION

Disturbances in PP2A methylation and one-carbon metabolism compromise Fyn distribution, neuritogenesis and APP regulation

Goce Taleski[1], Diana Schuhmacher[1], Henry Su[2], Jean-Marie Sontag[1] and Estelle Sontag[1]*

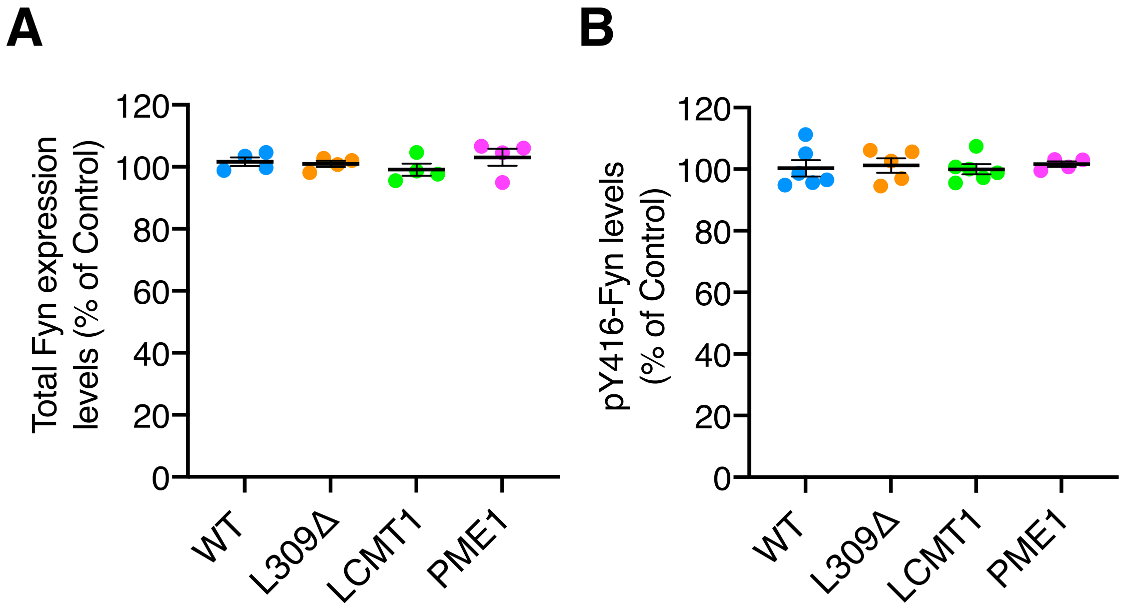

Supporting Figure 1: Quantification of total Fyn expression (**A**) and pY416-phosphorylated Fyn (**B**) levels in N2a cells stably expressing either WT PP2Ac, the L309Δ PP2Ac mutant, LCMT1, PME1, or empty vector (Control), as described in Fig. 2A. Total Fyn expression levels were determined in cell lysates after normalization for protein loading. pY416-Fyn levels were determined after normalizing the pSFK signal (apportioned to Fyn) for Fyn protein expression levels. Data are mean ± sem from n = 4-6 independent experiments and are expressed as percent of expression levels found in control cells. Under our experimental conditions, relative to control cells, there were no statistically significant differences in total Fyn protein expression levels or the net phosphorylation of Fyn among the cell lines examined, as determined by one-way ANOVA (Panel **A**: $F(4, 15) = 0.814$, $p = 0.536$); panel **B**: $F(4, 22) = 0.144$, $p = 0.964$).

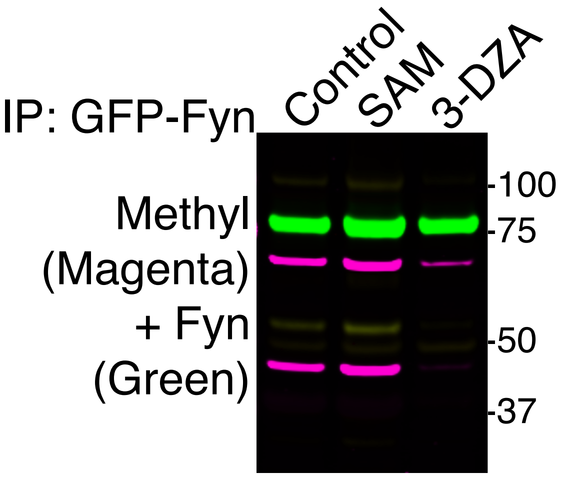

Supporting Figure 2: Analysis of Fyn lysine methylation state in N2a cells. GFP-Fyn expressing N2a cells were incubated for ~16 h with either 100 μM S-adenosylmethionine (SAM), 50 μM 3-Deazaadenosine (3-DZA) or vehicle (Control). GFP-Fyn immunoprecipitates were prepared from transfected N2a cells and analysed by Western blot using validated anti-methyl-lysine antibodies (Magenta), followed by reprobing with anti-Fyn antibodies (Green). Similar results were obtained in three separate assays.
